# Orexin/hypocretin modulation of neuroinflammation in a rodent model: implications for age‐related cognitive decline

**DOI:** 10.1002/alz.093087

**Published:** 2025-01-03

**Authors:** Marla A Frick, Brandy Lynn Somera, Jennifer L. Woodruff, Lawrence P. Reagan, Jim R. Fadel

**Affiliations:** ^1^ University of South Carolina School of Medicine, Columbia, SC USA; ^2^ Medical College of Georgia, Augusta, GA USA; ^3^ WJB Dorn VA Medical Center, Columbia, SC USA

## Abstract

**Background:**

The orexin/hypocretin neuropeptide system, primarily found in the lateral hypothalamus and perifornical region, modulates sleep, wakefulness, appetite, and cognitive function. One region with dense orexinergic projections is the basal forebrain (BF), which is the major source of acetylcholine in the neocortex and limbic structures such as the hippocampus. The basal forebrain cholinergic system mediates cognition and dysfunction is one of the key hallmarks of Alzheimer’s disease. We have previously shown significant reductions in orexin signaling and orexinergic innervation of cholinergic cells within the BF of aged rodents. Loss of orexin impairs cholinergic neurotransmission and cognitive function, but the mechanisms responsible for such deficits remain poorly understood. Recent evidence suggests neuroinflammation as a contributing factor to the pathogenesis of Alzheimer’s disease. It has been suggested that orexin may be neuroprotective, and we hypothesize that the age‐related loss of orexin neurons diminishes the brain’s anti‐inflammatory response, leading to basal forebrain cholinergic dysfunction.

**Method:**

Lentivirus mediated expression of Preproorexin antisense or sense was administered into the lateral hypothalamus of young adult (3 months; antisense) and aged (22‐26 months; sense), male and female Fisher 344/Brown Norway F1 hybrid rats. Three weeks later, a neuroinflammatory response was induced with an acute lipopolysaccharide (1 mg, intraperitoneal) challenge. 6 hours later, brains were removed and bilaterally dissected with one hemisphere post‐fixed in 4% paraformaldehyde for immunohistochemical analysis and one hemisphere frozen for cytokine analysis.

**Result:**

Lentivirus efficacy was verified using immunohistochemistry for GFP expression and changes in orexin expression within the lateral hypothalamus and terminal regions. There was no significant difference in total Iba‐1 in the basal forebrain, but there was a shift in activation state towards an activated, pro‐inflammatory, “M1” phenotype in the orexin antisense treated rats. In addition, there was an increase in IL‐6 and TNF‐alpha in the prefrontal cortex of orexin antisense treated male rats.

**Conclusion:**

Loss of orexin expression in aging may facilitate neuroinflammatory processes in key regions, such as the basal forebrain and prefrontal cortex, and thereby contribute to neurodegeneration and cognitive decline.

**Acknowledgements**: Supported by NIH R01 AG050518 and 2RF1 AG050518. Karly Pikel provided technical assistance.

